# A Public Opinion Propagation Model for Human-Made Disasters Considering Herd Behavior and Psychological Involvement

**DOI:** 10.3390/e28030303

**Published:** 2026-03-08

**Authors:** Yi Zhang, Ting Ni, Wanjie Tang

**Affiliations:** 1 Management School, Sichuan University of Science and Engineering, Zigong 643000, China; zhangyiyi@scu.edu.cn; 2 School of Environment and Civil Engineering, Chengdu University of Technology, Chengdu 610059, China; 3 West China School of Public Health, Sichuan University, Chengdu 610064, China; tangwanjie@scu.edu.cn

**Keywords:** human-made disasters, publicopinion propagation, psychological involvement, herd behavior, information diffusion dynamics, evolution of uncertainty

## Abstract

This study investigates the dynamics of information diffusion and uncertainty evolution in online public opinion systems under human-made disasters. A variant of the SIR model considering individual psychological involvement and group herd behavior is proposed. The theoretical analysis derives the propagation equilibrium points and the propagation threshold and further examines the stability of the system. The results indicate that the transmission rate, immunity rate, and herd behavior coefficient are key parameters influencing the dynamics of public opinion propagation. The simulation results validate the theoretical findings and provide a visualization of the sensitivity of the key parameters. Finally, an empirical case study is conducted to verify the effectiveness and applicability of the proposed model. The results indicate that controlling contact rate, reducing herd behavior, and lowering psychological involvement can effectively suppress opinion diffusion, with herd behavior and psychological involvement exerting a greater influence than contact rate on spreaders of the public opinion system. Consequently, mitigating public emotional resonance and herd effects constitutes an effective strategy for managing public opinion in human-made disasters, but reducing herd behavior makes the system relatively more uncertain compared with other scenarios. Finally, managerial implications for public opinion governance in human-made disasters are proposed. The findings enrich the theoretical system of information evolution modeling for complex social systems based on entropy and information theory, offer practical guidance for governments in developing scientific public opinion management strategies, and realize the transformation of public opinion systems from high-entropy disorder to low-entropy order.

## 1. Introduction

With the rapid development of Internet technologies and social media platforms, it is an important channel for the public to obtain information, express opinions, and participate in social issues through the Internet. Especially, after the sudden-onset disasters, a large amount of information spreads rapidly through online networks within a short period of time [1], forming a complex and dynamic online public opinion environment. The public opinion not only influences public risk perception and emotional attitudes [2] but may also affect governmental decision-making [3], social stability [4], and the effectiveness of emergency response [5]. In disaster classification systems, human-made disasters are recognized as a major category. In contrast to natural disasters, online public opinion in the context of human-made disasters exhibits distinctive characteristics. Such events are often accompanied by complex public discourse involving responsibility attribution, institutional deficiencies, moral evaluation, and conflicts of interest. These factors tend to stimulate high levels of individuals’ cognitive and emotional involvement, as well as passive herd behavior driven by social pressure and group identification mechanisms. Consequently, an in-depth investigation of the propagation mechanisms and evolutionary patterns of online public opinion in human-made disasters is of substantial theoretical significance and practical relevance for enhancing public crisis governance and social risk prevention capacities.

From the perspective of information theory, the online public opinion system can be regarded as a complex information system characterized by uncertainty. According to Shannon’s information theory [6], information entropy provides a quantitative measure for describing uncertainty and disorder in the process of information transmission. The intrinsic mechanisms of information diffusion are closely associated with entropy variation and the evolution of community organization [7]. In recent years, entropy-based approaches have gradually been extended to the study of online public opinion and social media dynamics. Chen et al. proposed an information entropy-based analysis method to address the uncertainty and fuzziness of online public opinion [8]. Yang et al. developed an entropy-based model for calculating and predicting public opinion popularity [9]. Tong et al. introduced a time-weighting mechanism and constructed models of local node entropy and global time-step entropy to effectively quantify the uncertainty and complexity of network topology [10]. These studies demonstrate that information entropy can effectively characterize the disorder, randomness, and instability of public opinion systems during their evolutionary processes. In the context of disasters, the rapid dissemination of information, emotions, and rumors significantly amplifies systemic uncertainty, driving the public opinion system toward a high-entropy, disordered state. Fu et al. introduced integrated intuitionistic fuzzy entropy in emergency public events, providing a more refined approach to handling complex public emotions [11]. Li et al. investigated sudden public health emergencies by applying the Brusselator model and the entropy method to examine the dissipative structure of online public opinion systems [12]. Son et al. employed statistical and predictive analyses to demonstrate that the greater the uncertainty of disaster-related tweets, the higher their entropy values [13]. Although existing studies have explored entropy-based analyses in disaster contexts, research that introduces entropy analysis methods into dynamic models of public opinion propagation during human-made disasters remains relatively limited.

In recent years, extensive research has been conducted on online public opinion propagation from the perspectives of information diffusion models, complex network theory, and game theory. Owing to the similarities between epidemic spreading and information diffusion, researchers have widely adopted epidemic dynamics to construct information propagation models. In 1985, Sudbury was among the first to employ the classical SIR model to investigate rumor spreading [14]. Due to their clear structure and strong interpretability, such models have been extensively applied to analyze public opinion diffusion processes [15], and many variants have subsequently been developed. Zhao et al. proposed a modified SIR-based public opinion propagation model by incorporating the influence of opinion leaders [16]. Yuan et al. introduced timeliness factors into the SIR model to investigate the polarization of public opinion in dynamic networks [17]. Xun et al. employed the SIR model to examine the temporal evolution of public opinion on social media platforms [18]. In addition, the classical SEIR model has also been widely used in information diffusion. Zhang investigated the control of online public opinion based on the SEIR framework and demonstrated its effectiveness in predicting and managing public opinion [19]. Yan et al. developed two time-delay negative information propagation models based on the SEIR framework [20]. Govindankutty et al. proposed an improved SEIR model by considering the tendency of misinformation propagation in online networks [21]. Furthermore, according to the characteristics of information diffusion, various factors have been incorporated into epidemic-based models, including media intervention [22], memory and forgetting mechanisms [23], hesitation behavior [24], and educational level [25], to characterize propagation patterns under different scenarios. These models offer valuable theoretical insights into the phased evolution, critical thresholds, and stability patterns of public opinion diffusion.

Many studies have emphasized the role of social network structures. Complex network theory suggests thatdiffusion outcomes often emerge from micro-level interaction patterns among individuals, including node centrality, community structure, and tie strength [15]. Wang et al. investigated the dynamic evolution patterns of public opinion and the influence of network characteristics on opinion distribution [26]. Geng et al. combined a multi-layer social network model with the SEIR framework to analyze information propagation [27]. This research perspective is highly consistent with innovation diffusion theory. Innovation diffusion theory posits that the spread of innovations is influenced not only by individual adoption decisions but also by the underlying social network structure. Factors such as individuals’ positions within the network, opinion leaders, and the overall communication structure play critical roles in innovation diffusion [28]. In such models, each node is assigned a state representing its adoption of an innovation, and model equations describe the temporal evolution of these states [29]. These models also analyze the balance between social-level diffusion dynamics and individuals’ perceived intrinsic benefits [30]. Innovation diffusion theory has been applied across numerous domains, including electric vehicle adoption [31] and artificial intelligence adoption [32]. Integrating complex network-based public opinion research with innovation diffusion theory can deepen our understanding of public opinion propagation models and strategy design, thereby providing a solid theoretical foundation for public opinion guidance and intervention.

In the study of disaster-related public opinion, research has primarily focused on the evolution patterns, influencing factors, and management strategies of public opinion during disasters. The disasters examined in these studies cover a wide range of events, such as typhoons, floods, public health crises, and food safety. For example, Wu et al. applied nonlinear programming models combined with machine learning methods to study public demand during typhoon disasters [33,34]. Li et al. investigated the development and evolution of online public opinion during rainstorm in China, providing important guidance for government actions [35,36,37]. Dai et al. conducted sentiment and topic analyses of public opinion during the MU5735 air crash by dividing the opinion cycle and classifying topics, highlighting the relationship between disaster-related topics and public sentiment [38]. Zhao et al. developed an information diffusion model for metro emergency based on bounded confidence models [39]. Zhang et al. constructed a sentiment classification model to analyze public reactions to food safety incidents [40]. During the COVID-19 pandemic, numerous studies focused on pandemic-related public opinion. Saricali et al. analyzed social media discussions regarding quarantine policies during the pandemic [41], while Jankowski et al. developed models to explore the interactions between epidemic spreading and opinion dynamics across multiple networks [42]. Jelodar et al. classified online comments related to COVID-19 and discussed the negative impacts of public opinion on both online platforms and real-world activities [43,44]. These studies generally focus on specific disaster cases and propose strategies for managing public opinion. For general emergency events, Chen et al. examined the intensity and direction of public opinion, as well as cognitive biases among online audiences, to support timely and targeted policy-making and public opinion management during emergencies [45,46]. Overall, the above research demonstrates that disaster-related public opinion has attracted widespread attention, and has yielded numerous valuable findings.

In existing research on information diffusion, although psychological involvement and herd behavior have received considerable attention, they are usually introduced and discussed separately and independently. Havitz et al. conceptualized psychological involvement as a motivational, arousal, or interest state that reflects an individual’s connection to a specific product or activity, grounded in their personality, cognition, motivation, and emotions [47,48,49]. Higher levels of psychological involvement can enhance individuals’ attention and enjoyment [50]. In 1967, Eagly examined individuals’ responses to different types of information under conditions of high and low involvement, finding that the level of involvement is a determining factor in responses to information [51]. Cameron conducted an experiment investigating the influence of involvement and limited prior knowledge on persuasive message components in investor relations [52]. Thornton emphasized the importance of educating children, health professionals, the public, and the media to promote active citizen participation in medical decision-making through high-quality health information [53]. Santosa et al. demonstrated that intrinsic motivation exerts a stronger positive influence on user involvement than situational motivation in the context of information-seeking activities [54]. In addition, Liu et al. proposed the PFDID model to characterize individual involvement driven by both subjective and objective motivations and verified its strong predictive performance [55]. Overall, these studies are largely confined to specific contexts and seldom examine the role of individual psychological involvement in information diffusion through propagation models.

Research on herd behavior has predominantly focused on financial markets. Investigating the relationship between information diffusion and herd behavior has become an important research direction [56]. Herd behavior was first observed in the field of financial investment, referring to the phenomenon in which investors tend to follow the actions of the majority in stock markets [57,58]. As research progressed, herd behavior has come to be defined more broadly as the tendency of individuals to conform to the opinions and behaviors of the majority under conditions of incomplete information or uncertainty [59,60]. Mezghani et al. analyzed contagion effects caused by herd behavior in financial markets [61]. Eguiluz et al. proposed a self-organizing model for information diffusion and group formation, applying it to describe herd behavior in financial markets and demonstrating that the interaction between information diffusion and herd effects can explain market crashes [58]. Delbari et al. examined collective behavior among social network users in information sharing and usage, suggesting that enhancing critical thinking and information literacy skills can reduce both the likelihood and manifestation of herd behavior [59]. Nian et al. based on the SI diffusion model, investigated how herd effects influence infection size in information spreading and demonstrated that herd behavior can induce abrupt changes in infection scale and significantly accelerate diffusion [62]. Yan et al. established an information competition model to analyze positive and negative information diffusion, and showed group influence intensifies herd behavior [63]. Although these studies provide valuable insights into herd behavior in information dissemination, systematic theoretical modeling and analysis of herd behavior in disaster contexts remain insufficient.

However, most models focus on characterizing public opinion propagation innatural disasters or public health events, and few studies examining public opinion in human-made disasters. During human-made disasters, the public often exhibits pronounced herd behavior and psychological involvement, which have significant impacts on the intensity, duration, and evolutionary patterns of public opinion but have not yet been systematically incorporated into existing models. Herd behavior tends to promote opinion consistency and reduce system uncertainty and information entropy, while high psychological involvement usually enhances information diversity and increases system entropy and disorder. The joint effect of these two behaviors impacts on evolutionary direction of the public opinion system. In the context of online public opinion propagation, when a particular viewpoint becomes dominant, other users are more likely to be influenced by the surrounding group and participate through sharing and commenting, thereby accelerating information diffusion and potentially triggering group polarization. Psychological involvement represents an internal state variable; in this study, it reflects the public’s attention, emotional investment, and perceived stake in an event. Evidently, highly involved individuals are more likely to actively seek information, engage in discussion, and continuously express their views, thus sustaining and amplifying the evolution of public opinion. In the context of human-made disasters, where events often involve sensitive issues such as safety responsibilities, conflicts of interest, and moral evaluation, the influence of herd behavior and involvement is particularly pronounced.

This study focuses on the propagation of online public opinion in human-made disasters. Based on epidemic dynamics theory, this study considers two dimensions: an internal dimension representing individuals’ cognitive and emotional involvement, and an external dimension reflecting herd behavior driven by social reinforcement and peer pressure. An improved SIR-based public opinion propagation model is developed by incorporating both herd behavior and involvement. By embedding the effects of herd behavior and psychological involvement levels into the transmission mechanism, the proposed model captures the influence of individual behavioral characteristics on the dynamics of public opinion diffusion and the evolution of system uncertainty. From the perspective of entropy and information theory, this study helps to reveal the change law of disorder in the evolution of public opinion. Subsequently, a theoretical analysis of the model’s equilibria, basic reproduction number, and stability conditions is conducted to elucidate how key parameters affect the scale and evolutionary trends of public opinion. Furthermore, social media data from a human-made disaster case are employed to estimate and validate the model parameters. Numerical simulations are then performed to examine the impacts of variations in herd behavior intensity and involvement levels on the propagation process under different scenarios. Finally, the results enhance the understanding of online public opinion evolution and provide a theoretical basis for governments and online platforms to formulate effective guidance strategies. The remainder of this paper is organized as follows: Section 2 develops the public opinion propagation model incorporating herd behavior and psychological involvement; Section 3 presents a dynamical analysis of the model; Section 4 and Section 5 conduct a simulation analysis and an empirical study; Section 6 presents the managerial implications; Section 7 and Section 8 discuss the results, summarize the study, and outline directions for future research.

## 2. Methods

In this section, individuals are categorized into three states to capture the characteristics of public opinion regarding human-made disasters and to identify the key factors influencing its dissemination. A differential dynamical system is then constructed to conduct the modeling analysis. Furthermore, the transmission threshold, equilibrium points, and the stability of these equilibria are rigorously investigated.

### 2.1. Public Opinion Influencing Factors

Unlike natural disasters, human-made disasters are generally traceable in terms of responsibility attribution. Such events often are closely related to public safety and human life, thereby easily triggering strong emotional resonance among the general public. Once exposed, they tend to attract widespread attention in a short period of time. Based on the characteristics of public opinion in the context of human-made disasters, this study identifies three key factors that play a dominant role in shaping public opinion related to human-made disasters.

Disaster severity. Human-made disasters result in severe consequences, including casualties, property losses and disruptions to daily life. Hence, there is a positive correlation between disaster severity and the dissemination of public opinion. Following the occurrence of a human-made disaster, the public has an urgent demand for all information about the incident. A higher degree of disaster severity leads to greater public attention and wider dissemination of public opinion. In other words, the severer the disaster, the higher the public attention it attracts and the broader the scope of public discourse it generates. On the contrary, if the human and economic losses are relatively slight, relevant public discussion will be scarce and tend to subside rapidly.

Psychological involvement. Involvement reflects the public’s level of attention, emotional investment, and perceived personal relevance to the disaster. Specifically, when individuals exhibit a high level of involvement in disaster, they tend to associate others’ adverse situations with their own potential experiences, thereby generating strong emotional resonance and heightened risk perception. Under such psychological conditions, individuals are more likely to immerse themselves in the disaster scenario and perceive similar events as personally relevant, which in turn motivates them to actively seek information, participate in discussions, and continuously express their views. In contrast, when involvement levels are relatively low, individuals tend to show little interest in disseminating related information.

Herd behavior. After the occurrence of disasters, online public opinion often spreads rapidly. The public are frequently exposed to large volumes of information before fully understanding the facts, and their judgments formed within a short period are therefore strongly influenced by the views of surrounding groups. When forming opinions, individuals tend to follow the prevailing trend, similar to sheep following a flock. In the context of human-made disasters, this tendency further promotes the widespread forwarding and diffusion of information.

### 2.2. Public Opinion Propagation Model

To analyze the evolution of online public opinion, the population in rumor propagation is analogously divided into three categories: ignorants, spreaders, and stiflers.

Ignorants refer to the proportion of netizens in the population who have never received any public opinion information. Ignorants have no ability to inhibit the spread of online public opinion, denoted as S(t).

Spreaders arethe proportion of netizens in the population who are aware of and actively disseminate public opinion information, denoted as I(t).

Stiflers arethe proportion of netizens in the populationwho are aware of the information but are not interested in further spreading it, denoted as R(t).

**Figure 1:**
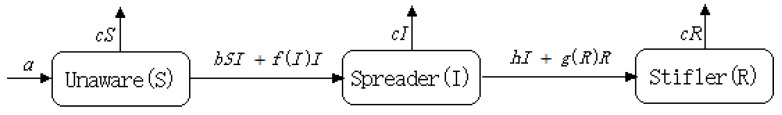
The state transitions among different groups.

The propagation of public opinion occurs through interactions among individuals. At any given time, each person may be in one of three states. It is further assumed that S(t), I(t) and R(t) are continuously differentiable function at any moment. Based on the classical SIR framework and the analysis above, the spread of public opinion in human-made disasters is influenced by disaster severity, psychological involvement, and herd behavior. The following sections analyze how these three factors are represented in the model. We construct a public opinion propagation model that incorporates all three human-made disaster factors. The propagation mechanism of the model is illustrated in Figure 1 and summarized as follows:

(1) When a disaster is severe, individuals in the ignorant population are more likely to actively seek to obtain relevant information, thereby increasing the probability of information transmission. At the same time, the decision of an individual to disseminate information is influenced by psychologicalinvolvement, which can further enhance the chance of sharing. Specifically, Human-made disasters often directly affect people’s livelihoods and are closely related to their daily lives, which can easily elicit empathy and strong emotional responses. Such involvement increases the likelihood that individuals will disseminate information. Consequently, high levels of involvement lead to a greater number of individuals converting into spreaders. Based on the above analysis, the proportion of individuals transitioning from ignorants to spreaders is directly influenced by both the severity of the disaster and the level of personal involvement. The transmission rate is given by b=λb(D,P), where D represents the severity of the disaster, P denotes the level of psychological involvement, and λ is the baseline transmission rate, i.e., the average influence that an informed individual exerts on others per unit time in the absence of other stimuli or reinforcing factors. Additionally, a denotes the proportion of new individuals entering the system per unit time, and c represents the removal rate, i.e., the proportion of individuals leaving the system per unit time. Parameters D, λ, a, c, are normalized to the interval [0,1]. Therefore, the rate of change of the ignorant population, expressed as a differential equation, is:
  (1) $$\frac{dS}{dt}=a-\lambda b(D,P)SI-cS$$

(2) Due to herd behavior, the group itself generates a stronger collective effect simply by virtue of its size. Therefore, the number of individuals in a group influences the growth of the group. Based on this principle, the increase in individuals within a group can be expressed as f(x)x, where f(x) s a function of group size x. This function can be used to quantify the influence of the group. Accordingly, under the effect of herd behavior, f(I)I reflects the increase in the number of individuals in the spreader group, and similarly for the stifler group, the increase in the number of stiflers is g(R)R. Spreaders convert to stiflers with probability h, which depends on involvement levels. If the public exhibits low involvement, their perception of the information changes, leading to a lack of interest in public opinion, and they cease to propagate the information, thus becoming stiflers. Therefore, the rate of change of the spreader population can be expressed as:
  (2) $$\frac{dI}{dt}=\lambda b(D,P)SI+f(I)I-hI-cI$$

The stiflers leave from the system at a constant rate c. From (2) and (3), there is

(3) $$\frac{dR}{dt}=g(R)R+hI-cR$$

Based on the above analysis, the public opinion model for human-made disasters can be expressed as follows:

(4) $$\left\{\begin{array}{l} \frac{dS}{dt}=a-bSI-cS\\ \frac{dI}{dt}=bSI+f(I)I-hI-cI\\ \frac{dR}{dt}=g(R)R+hI-cR \end{array}\right.$$

where parameters a, b, c, h are non-negative and take values within the interval [0, 1]. To analyze the model, it is necessary to know the explicit form of the function f(I) and g(R). However, since f(I) and g(R) are implicit function, we consider performing a Taylor expansion around a specific point, yielding:

$$f(x)=f(0)+f^{\prime }(0)x+\ldots +\frac{f^{\left(n\right)}(0)}{n!}x^{n}+o(y^{n})$$

To simplify the analysis, we retain only the linear component of the Taylor expansion. So

$$f(I)=f(0)+f(0)I=b_{0}+b_{1}I$$

$$g(R)=g(0)+g^{\prime }(0)R=c_{0}+c_{1}R$$

Accordingly, model (4) can be rewritten in the following form:

(5) $$\left\{\begin{array}{l} \frac{dS}{dt}=a-bSI-cS\\ \frac{dI}{dt}=bSI+(b_{0}+b_{1}I)I-hI-cI\\ \frac{dR}{dt}=(c_{0}+c_{1}R)R+hI-cR \end{array}\right.$$

Based on the definitions of the parameters, $b_{0},c_{0},b_{1},c_{1}$ are non-negative. The proposed model incorporates the effects of disaster severity, psychological involvement, and herd behavior. It is specifically designed based on the unique characteristics of human-made disasters, enabling a more accurate representation of the evolution of public opinion in response to such events. In the model, the parameter b is associated with both disaster severity and the level of involvement; the more severe the disaster and the higher the involvement, the greater the value of b. This implies that a larger number of individuals will transition into the spreaders. Additionally, $(b_{0}+b_{1}I)I$, $(c_{0}+c_{1}R)R$ reflect the influence of herd behavior. The larger the size of a group, the stronger the effect of the group itself, which in turn promotes further growth and development of the group. Themeanings of all parameters are provided in Table 1.

## 3. Findings

To explore the mechanisms through which various factors influence public opinion diffusion in human-made disasters, this section analyzes the fundamental properties of the model, including the information dissemination threshold and the stability of equilibrium points.

### 3.1. Equilibrium Point

To determine the equilibrium points, the right-hand sides of the three equations in model (5) are set to zero, yielding:

$$\left\{\begin{array}{l} a-bSI-cS=0\\ bSI+(b_{0}+b_{1}I)I-hI-cI=0\\ (c_{0}+c_{1}R)R+hI-cR=0 \end{array}\right.$$

Two types of equilibrium points can be obtained:

(1) Free equilibrium. When the number of spreaders is zero, no public opinion transmission occurs in the system. Such points are referred to as free equilibria. Obviously, $p_{0}=(\frac{a}{c},0,0),$ $p_{1}=(\frac{a}{c},0,\frac{c-c_{0}}{c_{1}})$ are the free equilibria of the model.

(2) Propagation equilibrium.

When the number of spreaders is nonzero, the above system of equations yields four nontrivial equilibrium points $p^{*}(S^{*},I^{*},R^{*})$, which can be obtained numerically using MATLAB R2016a. Owing to the complexity of their expressions, they are not listed here.

Since the free equilibrium represents the ideal state in which public opinion does not spread, this study mainly focuses on the propagation equilibrium.

It is very important to investigate the outbreak threshold of public opinion. In epidemiological studies, the basic reproduction number $R_{0}$ is a key threshold that determines whether an infectious disease can spread. When $R_{0}\geq 1$, an epidemic is expected to break out within a certain range; when $R_{0}<1$, disease transmission will gradually die out over time. Similarly, in human-made disasters, this study discusses the propagation threshold $R_{0}$ of online public opinion diffusion.

From the perspective of information entropy, this threshold also reflects the structural order of the public opinion system. When $R_{0}<1$, each spreader generates less than one new spreader on average, and diffusion gradually weakens. The proportions of different population groups converge toward a stable configuration dominated by stiflers or ignorants. In this case, the system transitions toward an ordered informational state, characterized by declining entropy and reduced structural uncertainty. In contrast, when $R_{0}\geq 1$, each spreader induces more than one new spreader, triggering sustained expansion of diffusion. The proportions of spreaders, stiflers, and ignorants become highly dynamic and competitive, leading the system into a structurally mixed and uncertain phase. Entropy increases as the distribution of group states becomes more balanced and dispersed, indicating an information-disordered diffusion state. Therefore, the propagation threshold not only determines whether public opinion will outbreak or decay, but also marks the critical boundary between informational orderand informational disorder in the public opinion system.

### 3.2. Propagation Threshold

Accordingly, the next-generation matrix method is adopted to compute the basic reproduction number of the proposed model [64]. First, the right-hand side of system (5) is decomposed into two components, the model can thus be rewritten as:

$$\left\{\begin{array}{l} \frac{dS}{dt}=F_{1}-V_{1}=0-(-a+bSI+cS)\\ \frac{dI}{dt}=F_{2}-V_{2}=bSI-[-(b_{0}+b_{1}I)I+hI+cI]\\ \frac{dR}{dt}=F_{3}-V_{3}=0-[-(c_{0}+c_{1}R)R-hI+cR] \end{array}\right.$$

Let $F={\left(F_{1},F_{2},F_{3}\right)}^{\prime }$, $V={\left(V_{1},V_{2},V_{3}\right)}^{\prime }$, then we can obtain

$$F=\left(\begin{array}{c} F_{1}\\ F_{2}\\ F_{3} \end{array}\right)=\left(\begin{array}{c} 0\\ bSI\\ 0 \end{array}\right)\;V=\left(\begin{array}{c} V_{1}\\ V_{2}\\ V_{3} \end{array}\right)=\left(\begin{array}{c} -a+bSI+cS\\ -(b_{0}+b_{1}I)I+hI+cI\\ -(c_{0}+c_{1}R)R-hI+cR \end{array}\right)$$

The compartment who do not know the information is S(t), hence $F_{2},F_{3},V_{2},V_{3}$ are considered, while $F_{1},V_{1}$ are not considered. By taking partial derivatives with respect to I and R, respectively, and substituting $p_{0}=(\frac{a}{c},0,0)$ into the expressions, the following simplified results can be obtained:

$$F^{\prime }=\left(\begin{array}{cc} \frac{\partial F_{2}}{\partial I}(P_{0}) & \frac{\partial F_{2}}{\partial R}(P_{0})\\ \frac{\partial F_{3}}{\partial I}(P_{0}) & \frac{\partial F_{3}}{\partial R}(P_{0}) \end{array}\right)=\left(\begin{array}{cc} \frac{ab}{c} & 0\\ 0 & 0 \end{array}\right)$$

$$V^{\prime }=\left(\begin{array}{cc} \frac{\partial V_{2}}{\partial I}(P_{0}) & \frac{\partial V_{2}}{\partial R}(P_{0})\\ \frac{\partial V_{3}}{\partial I}(P_{0}) & \frac{\partial V_{3}}{\partial R}(P_{0}) \end{array}\right)=\left(\begin{array}{cc} -b_{0}+h+c & 0\\ -h & -c_{0}+c \end{array}\right)$$

At the same time, the inverse matrix of *V*′ can be calculated,

$$V^{\prime -1}=\frac{1}{\left(-c_{0}+c)(-b_{0}+h+c\right)}\left(\begin{array}{cc} -c_{0}+c & 0\\ h & -b_{0}+h+c \end{array}\right)$$

$$\begin{array}{l} F^{\prime }V^{\prime -1}=\frac{1}{\left(-c_{0}+c)(-b_{0}+h+c\right)}\left(\begin{array}{cc} \frac{ab}{c} & 0\\ 0 & 0 \end{array}\right)\left(\begin{array}{cc} -c_{0}+c & 0\\ h & -b_{0}+h+c \end{array}\right)\\ \;\;\;=\frac{1}{\left(-c_{0}+c)(-b_{0}+h+c\right)}\left(\begin{array}{cc} \frac{ab(-c_{0}+c)}{c} & 0\\ 0 & 0 \end{array}\right) \end{array}$$

In the next-generation matrix approach, the basic reproduction number is defined as the spectral radius of the matrix $F^{\prime }V^{\prime -1}$ [64]. Accordingly, the basic reproduction number of the proposed model is $\rho (F^{\prime }V^{\prime -1})$. Therefore, the basic reproduction number, that is, the propagation threshold of this study, is defined as:

(6) $$R_{0}=\rho (F^{\prime }V^{\prime -1})=\frac{ab}{c(h+c-b_{0})}$$

The basic reproduction number obtained by substitution of $p_{1}=(\frac{a}{c},0,\frac{c-c_{0}}{c_{1}})$ is consistent with the one derived above. When $R_{0}<1$, the system has only the trivial equilibrium, and public opinion gradually fades from public attention over time. When $R_{0}\geq 1$, the model admits a nontrivial endemic equilibrium. Without effective intervention, public opinion will spread extensively throughout the system. According to Equation (6), the propagation threshold of public opinion is related to the inflow rate a, transmission rate b, outflow rate c, the conversion rate h from spreaders to stiflers, and the herd behavior parameter of spreaders, but is independent of the herd behavior coefficient of stiflers.

Parameter b represents the probability that an ignorant individual becomes a spreader, which depends on both the severity of the disaster and the level of psychological involvement. When other parameters are held constant, $R_{0}$ is positively correlated with b; that is, an increase in b leads to a corresponding increase in $R_{0}$. Therefore, when $R_{0}\geq 1$, controlling the spread of public opinion can be achieved by reducing the value of b. In practical terms, b can be decreased by limiting opportunities for ignorant individuals to contact spreaders in social networks and by alleviating public emotions, such as anxiety and concern. Parameter h describes the probability that an individual transitions from a spreader to a stifler, and it is also influenced by psychological involvement. Specifically, the lower the public’s involvement in a human-made disaster, the higher the probability that a spreader will become a stifler. Therefore, reducing involvement levels by alleviating anxiety and anger, through rational data-driven narratives to mitigate emotional resonance, as well as providing official communication channels to release tension, can help manage the spread of public opinion. $b_{0}$ represents the herd behavior coefficient of spreaders. A larger $b_{0}$ leads to a higher value of $R_{0}$, thereby increasing the risk of large-scale public opinion diffusion. Therefore, it is essential to release timely, authoritative, and diversified information to avoid single, emotion-driven expression and prevent the public from being influenced by one-sided viewpoints. In addition, avoiding excessive amplification of public opinion popularity, and clearly disclosing the progress of the event’s handling by relevant professional authorities can help curb active dissemination and group-aligned behaviors at the source.

### 3.3. Stability Analysis

Model (5) admits free equilibrium state $p_{0},p_{1}$. In the following, the stability of the model is examined at this equilibrium point.

**Theorem** **1.** *When* $\;0<R_{0}<1$ *, under condition* $-b_{0}+h+c>0,c-c_{0}>0$ *, the equilibrium point* $\;p_{0}=(\frac{a}{c},0,0,0)\;$ *of system (5) is locally asymptotically stable in the feasible region.*

**Proof.**  We consider the subsystem composed of these three equations. The Jacobian matrix of this subsystem at equilibrium point $\left(\frac{a}{c},0,0\right)$ is given by

$$J(p_{0})=\left(\begin{array}{ccc} -c & -\frac{ab}{c} & 0\\ 0 & \frac{ab}{c}+b_{0}-h-c & 0\\ 0 & h & c_{0}-c \end{array}\right)$$

Its characteristic equation is given by $\left|\lambda e-J(p_{0})\right|=0$, where *e* denotes the identity matrix. Specifically, the characteristic equation can be expressed as

$$(\lambda +c)[\lambda -(-b_{0}+h+c)(R_{0}-1)][\lambda -(c_{0}-c)]=0$$

The eigenvalues can therefore be obtained as

$$\lambda_{1}=-c,\lambda_{2}=(-b_{0}+h+c)(R_{0}-1),\lambda_{3}=c_{0}-c$$

When $0<R_{0}<1$, under condition $-b_{0}+h+c>0,c-c_{0}>0$,thus all the eigenvalues of are negative. According to the Routh–Hurwitz stability criterion [65], $E_{0}$ is a locally asymptotically stable equilibrium point of system (5). □

**Figure 2:**
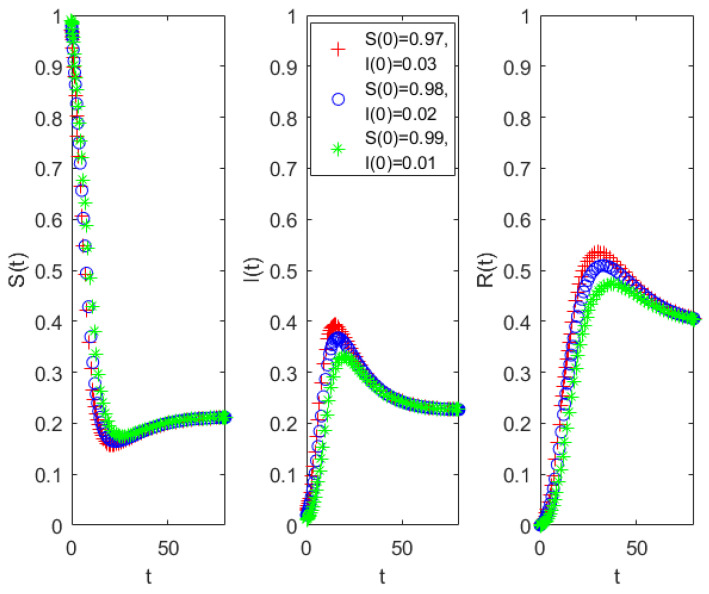
The evolution of the propagation process under varying initial conditions when $R_{0}>1$.

When the parameters are set as a=0.05, b=0.6, c=0.1, $b_{0}=0.1$, $b_{1}=0.1$, h=0.15, $c_{0}=0.01$, $c_{1}=0.01$, the basic reproduction number is calculated as $R_{0}=2>1$. Under this condition, System (5) admits a stable propagation equilibrium, which is illustrated by the simulation results in Figure 2. The numerical simulations indicate that although small perturbations in initial conditions lead to noticeable differences in the peak value of the infected population, all trajectories eventually converge to the same endemic equilibrium. This indicates that the equilibrium is asymptotically stable when $R_{0}>1$.Therefore, within a given propagation framework, even when spreaders are present in the system, their population eventually converges to a stable level. This implies that appropriate intervention and regulation measures can be implemented to guide the system toward a stable state and effectively control the diffusion of public opinion.

## 4. Simulation

To propose effective public opinion control and management strategies, numerical simulations were conducted to verify and visualize the theoretical analysis.

**Figure 3:**
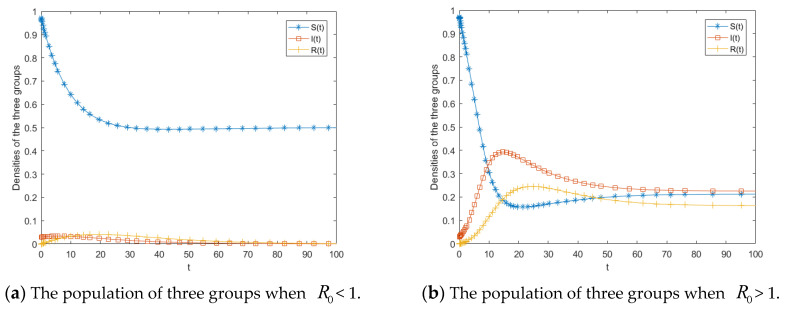
The population of three groups when $R_{0}$< 1, and $R_{0}$> 1.

First, we conducted numerical simulations to analyze the stability of the free equilibrium of system (5). The parameter values were set as a=0.05, b=0.2, c=0.1, $b_{0}=0.1$, $b_{1}=0.1$, h=0.15, $c_{0}=0.01$, $c_{1}=0.01$. The calculated propagation threshold *R*_0_ was 0.67 < 1, satisfying the conditions of Theorem 1. This verifies that system (5) is locally asymptotically stable at the free equilibrium. As illustrated in Figure 3a, public opinion eventually vanishes and does not exert a sustained impact on society. Under this condition, regardless of the initial values, the public opinion cannot persist or spread. Setting b = 0.6 while keeping other parameters unchanged yields $R_{0}$= 2 > 1. With the initial conditions b= (0.99,0.01,0), the simulation results shown in Figure 3b are obtained. At the early stage, the number of ignorants decreases rapidly, while the number of spreaders increases. Around t = 16, the spreaders reach a peak, after which their number gradually declines and eventually stabilizes at a constant level. As the propagation process evolves, spreaders progressively transform into stiflers, leading to a gradual increase in the number of stiflers. Meanwhile, due to the inflow of new ignorants into the system, the ignorant population exhibits a slight rebound. When $R_{0}$> 1, the numbers of spreaders and stiflers increase simultaneously, and the three populations ultimately converge to the endemic equilibrium. In this scenario, a proportion of individuals continue to disseminate public opinion information, resulting in a relatively high propagation intensity and broad social impact. Therefore, effective governmental intervention is required to control the spread of public opinion and mitigate the influence of public opinion. Overall, the simulation results in Figure 3 are consistent with the theoretical analysis, further demonstrating the critical role of the propagation threshold in determining the evolutionary dynamics of public opinion.

**Figure 4:**
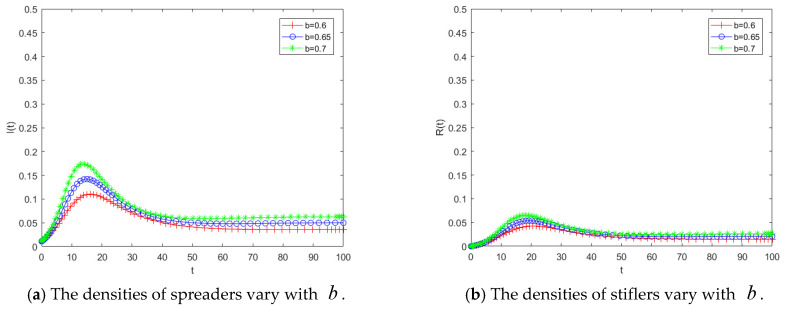
The densities of spreaders and stiflers vary with b.

**Figure 5:**
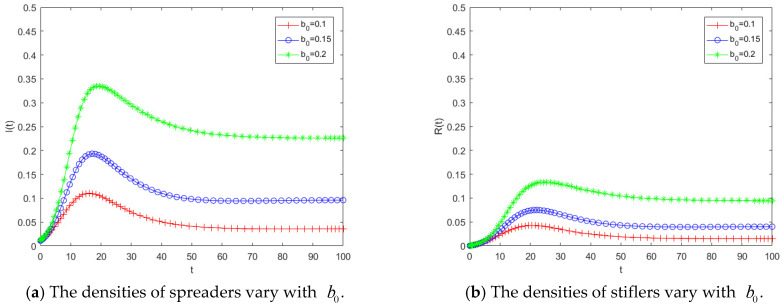
The densities of spreaders and stiflers vary with $b_{0}$.

**Figure 6:**
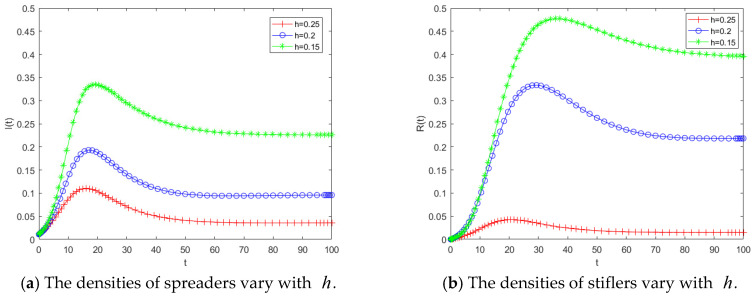
The densities of spreaders and stiflers vary with h.

From the expression of the propagation threshold, the key parameters include the transmission rate b, the psychological involvement parameter h, and the herd behavior coefficient $b_{0}$. Accordingly, Figure 4, Figure 5 and Figure 6 illustrate the effects of these three parameters on the propagation dynamics of public opinion in human-made disasters. For convenience in parameter adjustment, the baseline parameter values are set as a=0.05, b=0.2, c=0.1, $b_{0}=0.1$, $b_{1}=0.1$, h=0.25, $c_{0}=0.01$, $c_{1}=0.01$.

Figure 4 presents the temporal evolution of public opinion when the transmission rate b takes values of 0.6, 0.65, and 0.7. The results show that as b increases, the peak number of spreaders becomes higher, and the final number of stiflers also expands slightly. This indicates that a larger transmission rate enhances both the intensity and the overall impact of public opinion propagation. However, judging from the magnitude of variation in the curves, although increasing b promotes the spread of public opinion, the overall change remains relatively moderate. These findings suggest that reducing the transmission rate can contribute to controlling public opinion dissemination; nevertheless, the propagation dynamics are not highly sensitive to small changes in the transmission rate. Therefore, effective control through this pathway requires substantial efforts to significantly decrease the transmission rate in order to achieve desirable outcomes.

Figure 5 illustrates the variations in the densities of spreaders and stiflers under different values of the herd behavior coefficient $b_{0}$. Overall, a larger $b_{0}$ leads to greater propagation intensity and a wider diffusion range of public opinion. Figure 5a depicts the temporal evolution of the spreader density as $b_{0}$ varies. Under the same magnitude of parameter change, compared with the transmission rate, an increase in $b_{0}$ results in a more pronounced rise in the number of spreaders, indicating that higher herd behavior significantly accelerates the propagation process. This finding further demonstrates that public opinion dynamics are highly sensitive to the herd behavior coefficient; even small variations in $b_{0}$ can induce marked changes in the number of spreaders. Figure 5b shows the evolution of the stifler density over time for different values of $b_{0}$. As $b_{0}$ increases, the final number of stiflers also rises, suggesting that a larger proportion of individuals are ultimately influenced by the public opinion. According to the analytical expression of the propagation threshold, an increase in $b_{0}$, enlarges $R_{0}$, making it more likely for the system to enter the region where $R_{0}$ > 1, thereby triggering large-scale propagation. This theoretical implication is consistent with the simulation results shown in Figure 5a, where the number of spreaders increases rapidly as $b_{0}$ grows. Hence, the numerical simulations provide empirical support for the threshold conclusions derived from the theoretical analysis. The results indicate that public opinion propagation is highly sensitive to herd behavior effects. Even a modest enhancement in herd behavior can substantially amplify the scale of dissemination. Therefore, compared with solely reducing the transmission rate, mitigating the influence of herd behavior may represent a more effective governance strategy for controlling public opinion diffusion.

Figure 6 presents the temporal evolution of spreaders and stiflers when theimmunity rate h takes values of 0.15, 0.20, and 0.25. As shown in Figure 6a, when h is relatively small, the peak of the spreader curve is higher and the growth rate is faster. A stronger level of psychological involvement implies that individuals are more reluctant to cease dissemination, thereby hindering the transition from spreaders to stiflers. Figure 6b further indicates that when h decreases, the final number of stiflers at the end of the propagation process increases, suggesting that a larger proportion of the population is ultimately affected by the public opinion event. This observation is consistent with the threshold analysis, as h decreases, the threshold $R_{0}$ increases, leading to a more pronounced amplification effect in disaster-related public opinion propagation. Comparing these results with the variation in b in Figure 5, it can be observed that both parameters exert similar influences on the spreader population. This is because the two coefficients occupy analogous positions in the model structure. However, their effects on the stiflers population differ. The number of stiflers is more sensitive to changes in psychologicalinvolvement, implying that even a small increase in h can significantly reduce the overall impact range of public opinion. The simulation results demonstrate that psychological involvement serves as a critical regulatory variable in shaping the scale of disaster-related public opinion dissemination. When the h is low, individuals remain in a prolonged state of heightened emotional engagement and are unwilling to withdraw from the dissemination process, thereby extending the duration of propagation and enlarging the final scope of influence. Therefore, public opinion governance should not rely solely on limiting information transmission or mitigating herd behavior; it is equally important to reduce excessive psychological involvement among the public, as doing so can substantially diminish the overall impact of public opinion events.

## 5. Case Study

In this section, the model parameters were first estimated based on the case study data. The proposed model was then applied to analyze the case, and the simulation results were compared with those of the classical SIR model. Finally, a sensitivity analysis of the parameters was conducted to assess the model’s response to variations in key parameters, providing a basis for recommending public opinion management strategies.

### 5.1. Data Collection and Analysis

On 22 May 2021, a severe public safety incident occurred near the Labor Park in Dalian, Liaoning Province, China. The suspect motivated by social revenge following a failed investment, deliberately ran a red light and struck multiple pedestrians, causing five fatalities and five injuries. He fled the scene but was apprehended later the same day.

The incident immediately drew widespread attention online. Videos posted by netizens were rapidly reposted by influential social media accounts, and trending topics such as “Multiple pedestrians hit at Dalian Labor Park” and “Dalian Labor Park car accident” emerged. Public sentiment was dominated by outrage toward the perpetrator. After the official investigation confirmed the suspect’s deliberate intent, negative emotions further intensified and polarized across social media. By 23 May, the hashtag #Dalian Car Accident Suspect Motivated By Revenge# went viral, reflecting a rapid surge in online attention and driving the public opinion toward a peak. This study applies the proposed model to analyze this case. By examining the dynamics of this case, we aim to identify general patterns in public opinion diffusion and provide insights for effective management and guidance in human-made disaster.

Based on the event timeline, the key factors influencing the diffusion of public opinion are summarized as follows:

Disaster severity. The incident resulted in four immediate fatalities and one additional death after emergency treatment, posing a severe threat to public safety. The investigation team classified it as a major accident. To quantify the severity of the disaster, we assign a score of 1 for high severity and 0 for low severity. Therefore, the severity coefficient of this incident is set as D = 1.

Psychological involvement. The perpetrator deliberately struck pedestrians on the sidewalk in this incident, an act of extreme severity that posed a serious threat to public safety. The event was initially disseminated through videos posted by netizens, which provided highly vivid and direct visual information, conveying intense emotional impact due to the tragic scene. Moreover, the incident occurred in a central area of Dalian, prompting individuals to imagine themselves in similar situations and perceive the potential risk to their own safety. Consequently, the public exhibited a high level of attention to the event, strong emotional investment, and heightened perceived personal relevance, motivating them to actively follow, engage with, and share information related to the disaster.

Herd behavior. Before the official investigation clarified the cause, online discussions speculated widely about the perpetrator’s motives, ranging from drunk driving, brake failure, and sudden medical conditions to even personal issues such as marital problems. Such uncertainty and curiosity drove intense online attention and rapid dissemination of information. Many netizens followed prevailing opinions, actively discussing and sharing these speculative narratives, illustrating the role of herd behavior, where individuals’ actions and judgments are influenced by the perceived views of the majority.

**Figure 7:**
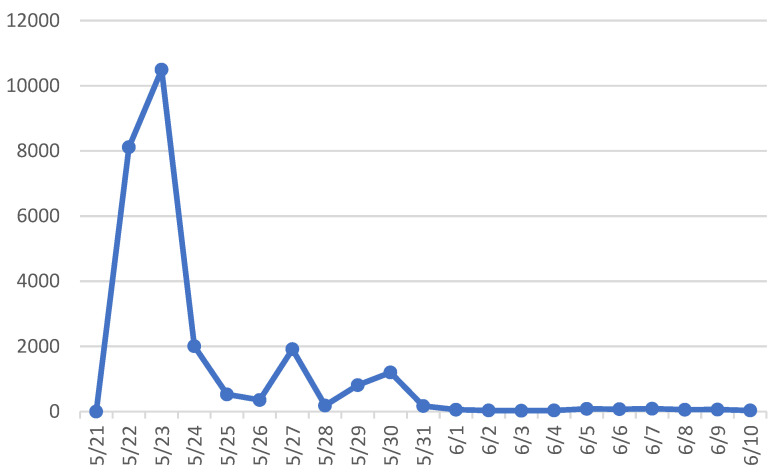
Number of Weibo posts over time in the case study.

To investigate the propagation characteristics and dynamics of the case, we collected online data from Sina Weibo, a widely used Chinese microblogging platform that enables real-time information sharing and user interaction, to validate the proposed model. Using the keywords “Dalian car accident” and “Dalian hitting pedestrians”, we retrieved posts from 21 May to 10 June, resulting in a total of 26,357 Weibo entries. The collected data included user nickname, user ID, posting time, and posting content. Daily counts of public opinion-related posts were then calculated, as shown in Table 2. Finally, theevolution of daily post volumes was visualized, with time on the x-axis and the daily number of Weibo posts on the y-axis, as illustrated in Figure 7.

Based on the trend depicted in Figure 7, the full evolution of the incident is analyzed in conjunction with the actual events. At noon on 22 May, multiple netizens posted video reports of a severe traffic accident at the Laodong Park, which were promptly reshared and disseminated by several online influencers. This led to the emergence of trending topics such as ‘Car hits multiple pedestrians at Dalian Laodong Park’ and ‘Dalian Laodong Park traffic accident’. However, the incident did not reach a significant peak in online attention on the same day. On the afternoon of 23 May, the official WeChat account Dalian Release issued a statement indicating that the suspect Liu, unable to accept his investment failure and having lost faith in life, developed a psychologically driven desire to retaliate against society. Following this official disclosure, the online dissemination and public attention to the incident increased sharply, propelling the event to its peak in public opinion. Subsequently, the attention gradually declined, and by 26 May, public engagement had substantially decreased. On the evening of 27 May, the official WeChat account of the Dalian Procuratorate announced that on 26 May 2021, the authority approved the arrest of Liu, the suspect in the case. This announcement was subsequently reposted by several prominent media organizations under the hashtag #Dalian BMW hits pedestrians, killing 5; suspect arrested#, triggering a renewed wave of public attention. From the perspective of the overall evolutionary process, human-made disasters exhibit high destructiveness and evoke strong public emotions, which can easily lead public opinion crises during online dissemination.

To estimate the parameters of the public opinion dissemination model, the propagation process of the case was tracked in real time, and detailed statistics on the states and transitions of all users within the system were conducted. Given the large volume of Weibo data and the discontinuity of activity among many Weibo users, direct parameter extraction was challenging. Therefore, a sampling-based approach was employed, whereby user state distributions were calculated from the sample of the collected data. To ensure both continuity and activity of users, the dataset was filtered to retain only those who had published at least three Weibo posts, reducing the database to a more manageable size. From this filtered pool, a random sample of 380 users was selected, yielding a total of 1539 Weibo posts. As shown in Figure 3, by approximately 1 June, the online dissemination of public opinion regarding the incident had largely ceased. Therefore, using the sampled dataset, the number of active users and posts was compiled for the period from 22 May to 1 June. During this interval, the 380 sampled users generated 1467 posts. These statistical results were then mapped to the parameters of the public opinion dissemination model, producing the parameter set summarized in Table 3. This approach allows for a representative estimation of model parameters while accounting for user continuity and activity in the observed system.

Considering the timing of the disaster, the model defines the number of individuals prior to 14:00 on 22 May as the initial number of spreaders, which was 4. Therefore, at the initial stage of the propagation, there were a total of 376 ignorants in the system. As shown in Figure 2, the incident occurred on 22 May, with the number of spreaders reaching its peak on 23 May, followed by a gradual decline. The following part presents the calculation of the parameters in Model (5).

During 22–23 May, the number of spreaders among the unaware population was increasing. On 22 May, 149 spreaders emerged, leaving 376 − 149 = 227 ignorant individuals. On 23 May, the number of spreaders increased to 277, meaning that 277 − 149 =128 new spreaders were added. Therefore, the average transmission rate over these two days was directly calculated as b = (149/376 + 128/227)/2 = 0.5. From 24 May to 27 May, the number of spreaders exhibited a declining trend. To estimate the parameter h, the average number of spreaders from 24 May to 1 June was calculated as 76, with all users participating in the dissemination process during this period. Accordingly, h = (277 − 76)/380 = 0.53. The initial values for each group can also be derived from the data presented in Table 3, $S_{0}$ = 376/380 = 0.987, $I_{0}$ = 4/380 = 0.013, and the parameters for this case are summarized in Table 4.

### 5.2. Comparative Analysis

First, without considering herd behavior, the model is reduced to a classical SIR model. The parameters listed in Table 4 were incorporated into the classical dissemination model. During the sampling of Weibo posts, it was observed that many participating users continuously followed the incident, and the number of individuals leaving the system was relatively small. Additionally, in reference [66], the outflow rate in the SIR model was set to 0.01; therefore, c = 0.01 was adopted. In the era of social media, information is continuously reposted and reported, attracting more netizens who were originally not involved to enter the discussion space. As a result, the number of netizens entering the system should be greater than those leaving it. Therefore, we set a = 0.05. Simulations were conducted in the MATLAB R2016a to examine the changes in the populations of ignorants, spreaders, and stiflers. The simulation results are presented in Figure 4.

**Figure 8:**
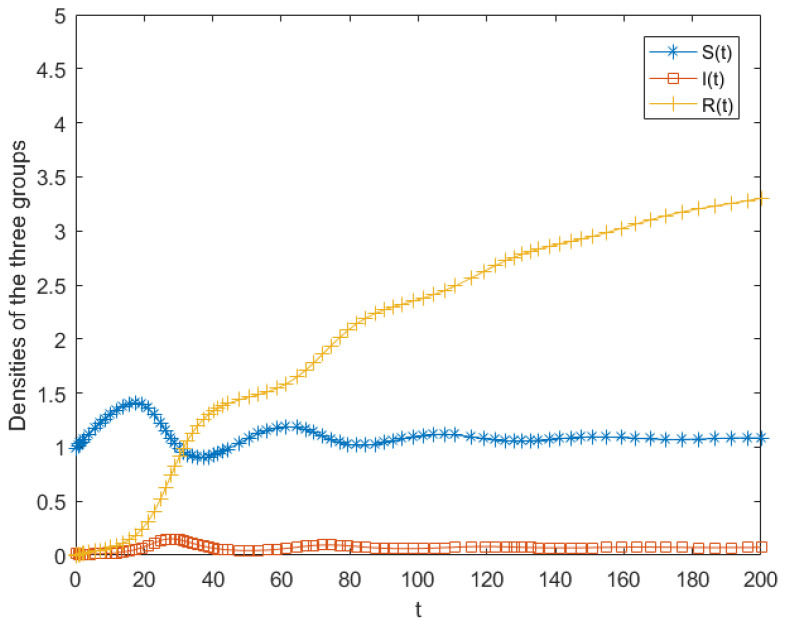
Densities of different groups without considering herd behavior in case study.

As shown in Figure 8, the number of spreaders reaches its maximum at approximately t = 15, with a peak density of around 0.2. In comparison with the actual event, the spreader population in the real case reached its peak earlier, with a peak value of 277/380 = 0.729. This indicates that the simulation presented in Figure 8 underestimates both the intensity and the progression of dissemination. The discrepancy can be attributed to the model’s omission of herding behavior.

From the evolution of the case study, it can be observed that once the cause of the incident was clarified and the suspect was apprehended, public attention rapidly declined, and the incident no longer attracted widespread interest. Therefore, in this case study, we focus on the herd behavior among spreaders. Between 22 May and 23 May, the number of spreaders increased by 128 individuals. Among these, some were generated by the first part of $\frac{dI}{dt}$ in the model, primarily influenced by transmission rate, while the remainder can be attributed to the herd behavior.

Using Weibo data, it is not possible to distinguish whether an individual shares information due to rational judgment or simply due to observing that many others have shared it; therefore, the proportion of herd-driven dissemination cannot be directly observed from real-world data. Classic research on group pressure by Asch found that even in judgment tasks with clear objective answers, individuals changed their original decisions to conform to the majority in a substantial proportion of cases, with erroneous conforming judgments accounting for approximately one-third of all responses [67]. This result indicates that herd behavior exerts a significant influence on individual decision-making. Furthermore, social influence mechanisms also play an important role in online environments. In an online social network experiment, Damon found that among participants receiving three signals, 40% revisited the forum at least once, demonstrating that social reinforcement significantly affects the individual’s adopted behaviors [68]. Although these experiments were not conducted specifically in the context of information dissemination, they provide important psychological evidence for understanding how individuals may adjust their sharing behavior in response to majority actions during online public opinion propagation. Therefore, in highly developed social media environments, and given that disaster-related public opinion exhibits clear herd characteristics, it is theoretically reasonable to treat herd effects as a driver of similar importance to direct information exposure. Therefore, we made a relatively neutral assumption that half of the 128 individuals were influenced by herd behavior. Under this assumption, the two related parameters $b_{0},\;b_{1}$ satisfy $(b_{0}+b_{1}\frac{149}{376})\frac{149}{376}=\frac{64}{380}$, that is, $b_{0}+0.4b_{1}=0.42$, Let $b_{1}=0.1$, then $b_{0}=0.34$ can be obtained. Regarding the herd behavior among stiflers, the decrease in spreaders did not begin until 24 May. Therefore, from this date, we calculated the daily reduction in the number of spreaders. By 1 June, excluding days with an increase in spreaders, a total of nine days were considered, and the average daily increase in stiflers was 45 individuals. This increase is primarily driven by factors such as involvement and attitude changes. The average daily variation is relatively small, and from the perspective of incident management, these changes mainly reflect public satisfaction with the handling of the event. Consequently, herding behavior among stiflers is not considered in this case.

By substituting all estimated parameters into Model (5), a simulation analysis of the proposed public opinion dissemination model for human-made disaster events was conducted. The temporal variations in the populations of unaware individuals, spreaders, and stiflers under the case scenario were thereby obtained. The simulation results are presented in Figure 8.

**Figure 9:**
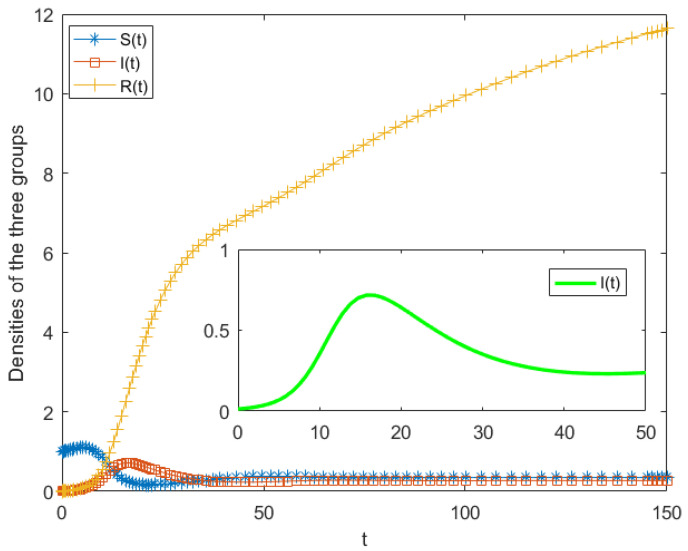
Densities of different groups in case study.

Figure 9 illustrates the density variations of different population groups in case study. As shown in the figure, public opinion dissemination reached its peak approximately 12 h after the occurrence of the incident, as reflected by the spreader population attaining a maximum density of about 0.7. Subsequently, the number of spreaders declined and gradually stabilized around t = 50. This trend is consistent with the actual Weibo data, which indicate that online public attention to the incident diminished to near zero approximately three days after its occurrence. Over time, as ignorants transitioned into spreaders, the population of ignorants decreased rapidly and eventually approached zero. This suggests that the incident spread rapidly through the online network, influencing a large number of previously uninformed individuals within a short period. Meanwhile, as both ignorants and spreaders converted into stiflers, the stifler population exhibited a continuous upward trend before quickly reaching a stable state. Since this model considers that new ignorants enter the system per unit time, the values on the y-axis are no longer fixed proportions of the total population. The total population increases over time. Among them, the number of stiflers is relatively large at the end, indicating that a large number of incoming ignorant individuals were influenced by the public opinion, and many people have transmitted the information. During the dissemination process, all three groupsexperienced rapid changes, indicating that online public opinion related to the incident underwent an explosive diffusion in a short time. Such rapid propagation substantially increased the difficulty of governmental monitoring and intervention. In this case, public discourse was initially dominated by sympathy for the victims and condemnation of the perpetrator, accompanied by speculation regarding the motive. With the gradual disclosure of official information, online sentiment shifted toward intensified criticism of the offender, together with refutation of previously widespread misinformation. Therefore, online public opinion triggered by human-made disaster should be closely monitored and proactively managed, with timely and effective response mechanisms implemented to mitigate potential risks.

The classical SIR model and the proposed model were applied to simulate the case. The results indicate that the classical SIR model significantly underestimates the intensity and speed of public opinion dissemination. In particular, it fails to accurately capture the rapid growth and high peak of the spreader population observed in the real event. In contrast, the proposed model provides a more realistic representation of the dissemination process, closely reproducing the temporal evolution and peak characteristics of online public attention. The simulation results show good agreement with the empirical Weibo data in terms of both propagation trend and magnitude. This demonstrates that the proposed model exhibits superior performance in describing public opinion dynamics associated with human-caused disaster events. Overall, the comparative results confirm the effectiveness and practical applicability of the proposed model, highlighting its advantages in capturing herd behavior and psychological involvement in online dissemination.

### 5.3. Control Strategy Analysis

Based on the preceding theoretical analysis and simulation results, we conclude that the transmission coefficient, herd behavior coefficient, and immunity coefficient play crucial roles in the propagation of public opinion during human-made disasters. The transmission coefficient is directly related to the contact rate and the severity of the disaster. The herd behavior coefficient reflects the influence of conformity behavior, while the immunity coefficient is primarily associated with individuals’ levels of psychological involvement. In the following section, we compare the variations in public opinion evolution under adjustments to these parameters in order to evaluate the advantages and limitations of different governance strategies.

To analyze the control strategies for human-made disasters, four groups ofscenarios were designed in this section. The specific parameter settings are presented in Table 5.

**Figure 10:**
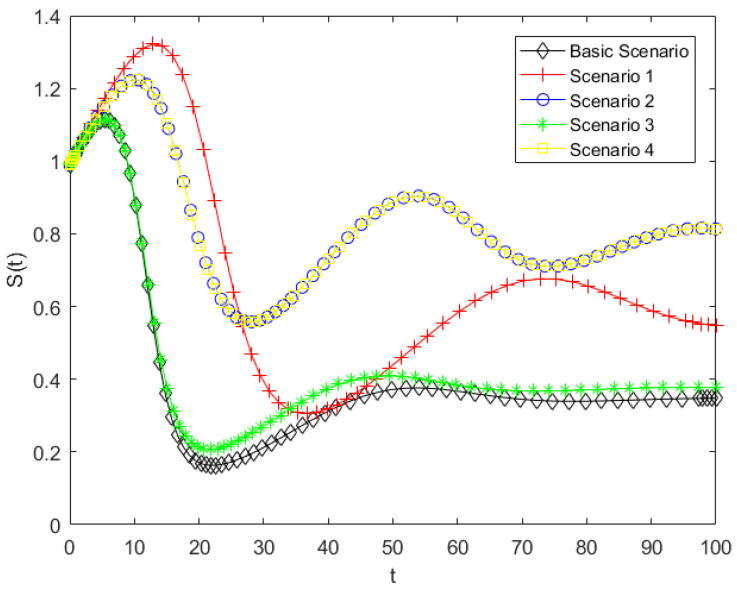
Density of ignorants under different scenarios.

**Figure 11:**
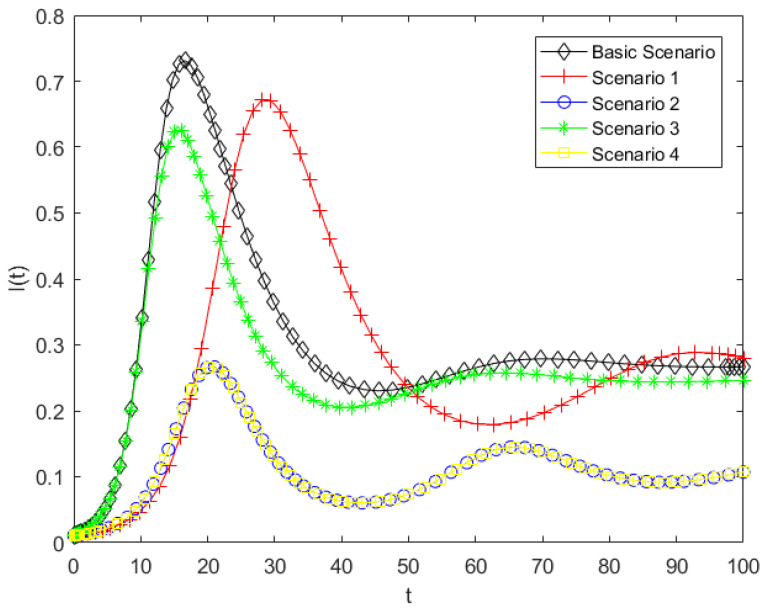
Density of spreaders under different scenarios.

**Figure 12:**
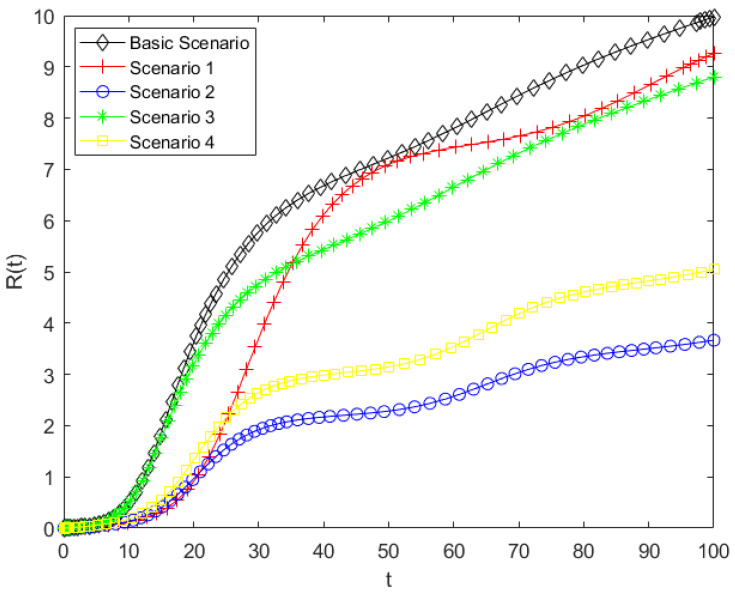
Density of stiflers under different scenarios.

By substituting the parameters listed in Table 5 into Model (5), a simulation analysis of the public opinion dissemination model for the case was conducted. This allowed the variations in the populations of ignorants, spreaders, and stiflers under different scenarios to be obtained. The simulations further explored the effects of various intervention strategies on public behavior and state transitions, providing insight into the optimal approaches for managing online public opinion during disaster events. The corresponding simulation results are presented in Figure 10, Figure 11 and Figure 12.

As shown in Figure 10, compared with the baseline scenario, the other four scenarios represent different forms of government intervention, all of which have varying effects on the dynamics of ignorant individuals. The final value of S(t) represents the proportion of individuals who have never been exposed to public opinion. As shown in the results, this proportion significantly increases under Scenarios1, 2, and 4, indicating that the number of individuals who have been exposed to public opinion is correspondingly reduced. In particular, under Scenarios2 and 4, the decrease in the density of ignorants is substantially mitigated, resulting in the largest remaining proportion at the end of the simulation. In contrast, Scenario3 exhibits the weakest influence, with only a marginal impact on the evolution of ignorant individuals. These results indicate that Scenarios2 and 4 are the most effective in reducing the scope of influence of online public opinion., whereas Scenario3 performs the poorest. According to the meanings of parameters $b_{0}$, $b_{1}$, b and h, reducing the parameter of herd behavior and promoting the transition from spreaders to stiflers are the most effective strategies for mitigating the impact range of online public opinion, and reducing the contact rate has a certain effect; however, it is far less effective than controlling herding behavior and psychological involvement. In the Dalian intentional vehicle-ramming incident motivated by retaliation against society, related videos and unverified information spread rapidly across social media platforms during the early stage of the event. A large number of netizens engaged in reposting and commenting, which rapidly expanded the scope of information dissemination related to the incident. This emotional involvement and collective herd behavior directly influence the immunity coefficient h and the herd behavior coefficient $b_{0}$ in the model. Therefore, in human-made disaster events, curbing irrational herd behavior and guiding the public toward rational participation can effectively reduce the transition of ignorants into spreaders, thereby limiting the overall scope of public opinion diffusion.

As shown in Figure 11, compared with the baseline scenario, all alternative scenarios result in a lower peak of the spreader population. In Scenarios 2 and 4, spreaders exhibit the lowest peak and slowest growth. This indicates that Scenarios 2 and 4 are most effective in limiting the spreaders, Scenario 3 has a moderate effect, and Scenario 1 is the least effective. These results indicate that decreasing the herd behavior coefficient and increasing the probability of spreaders converting into stiflers have a significant inhibitory effect on the growth of spreaders. Therefore, reducing herd behavior and lowering individual psychological involvement are key factors in inhibiting the growth of spreaders. In human-made disasters, public opinion typically exhibits a pattern of rapid escalation within a short period of time. In case study, the public often engages in concentrated discussion and continuous attention within a highly emotional atmosphere, leading the number of spreaders to reach a peak in a very short time. This phenomenon is highly consistent with the rapid formation of the peak number of spreaders observed in the model. In reality, the timely release of authoritative information, transparent disclosure of disasters progress, and clarification of misinformation can help reduce the public’s level of psychological involvement, thereby facilitating the transition of some spreaders into stiflers. This can delay the emergence of the transmission peak and reduce its magnitude. These findings suggest that, compared with merely restricting information contact channels, regulating individual psychological mechanisms and herd tendencies plays a more critical role in suppressing the intensity of public opinion diffusion.

As shown in Figure 12, consistent with the results in the previous two figures, the final number of stiflers is significantly reduced in Scenarios 2 and 4, while Scenarios 1 and 3 show only minor changes. Among the measures that decrease the stifler population, Scenario 2 is the most effective, followed by Scenario 4, confirming that both types of interventions can effectively limit the scope of public opinion. The pronounced effect of Scenario 2 highlights the critical role of mitigating herd behavior in public opinion management. Since this model considers that new ignorants enter the system per unit time, the total population is not fixed. Therefore, the values on the vertical axis represent the cumulative number of stiflers over time, which can exceed 1. A higher number of stiflers indicates that more people have been influenced by the public opinion, meaning that a larger portion of the public has ceased to propagate the information after being affected. This indicates that increasing the transition rate from spreaders to stiflers and reducing herd behavior in the model can gradually drive the system from a highly active state to a stable state. From the perspective of system evolution, such interventions not only control the scale of dissemination but also suppress the continued accumulation of structural uncertainty in the public opinion system, thereby preventing the system from evolving toward a high-entropy and disordered state. Therefore, in managing human-made disasters, mitigating herd behavior and reducing psychological involvement are more effective governance strategies than simply lowering the contact rate.

Although the proportion of spreaders can directly reflect the intensity and scale of public opinion diffusion, it only captures the state of a single group and cannot comprehensively describe the structural characteristics of the system. Focusing solely on spreaders may underestimate potential risks. In the propagation system, when the proportions of the three groups are similar, the system is in a state of structural competition and dynamic interplay, making it more sensitive to external disturbances. For policymakers, this may require more timely and precise interventions. Accordingly, a system-level information entropy is defined, and analyzing it together with the proportion of spreaders helps identify high-risk, disordered stages.

**Figure 13:**
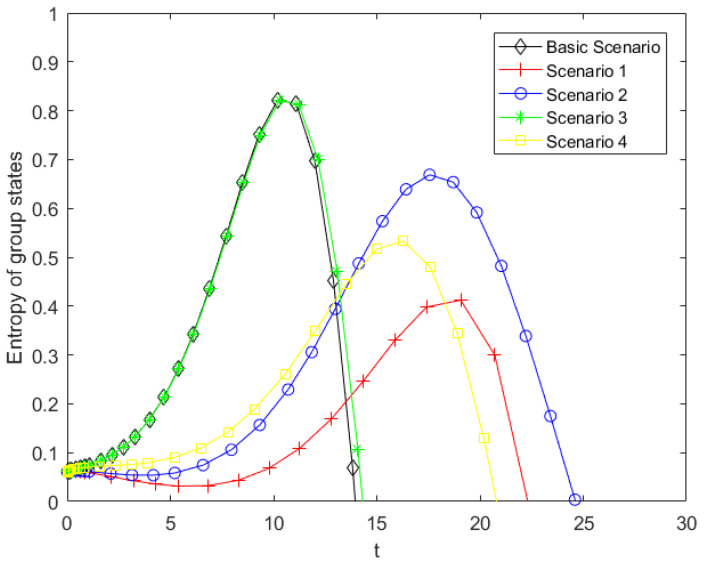
Entropy of group states under different scenarios.

To reflect measure the overall uncertainty of the system and the equilibrium degree of group structure distribution, a ternary information entropy composed of the proportions of the ignorants, spreaders, and stiflers is defined, that is H=-SlnS-IlnI-RlnR. Figure 13 illustrates the temporal evolution of this entropy. The entropy trajectory exhibits a typical nonlinear pattern characterized by an initial increase followed by a subsequent decline. In the early stage, the unaware individuals dominate the system, resulting in a relatively homogeneous population structure and low uncertainty; consequently, the entropy remains at a low level. As the information spreads, the number of spreaders increases and stiflers begin to emerge, leading the system into a competitive phase between diffusion and suppression. The entropy reaches its peak when the structural uncertainty of the system is maximized and the system structure is in a state of maximum uncertainty. At this moment, the system is not only statistically disordered but also structurally unstable, as no single state dominates the evolution trajectory, and this indicates that there is a strong game relationship and evolutionary uncertainty within the system. From a governance perspective, a high-entropy state means that the public state is highly fragmented, which makes it more difficult for authoritative information to quickly consolidate consensus. Therefore, the entropy peak can be a key structural risk point, representing the highest level of uncertainty faced by policymakers. In this context, the effectiveness of interventions becomes more sensitive to timing and credibility, thereby increasing the complexity of governance. Thereafter, as one group gradually dominates, the system transitions toward a more concentrated structure, and entropy declines, indicating a shift from a disordered competitive state to a relatively ordered and stable state.

However, significant differences are observed across governance scenarios. In the baseline scenario, the entropy rapidly reaches its peak, suggesting that in the absence of effective intervention, the population quickly evolves into a highly mixed state. This rapid chaotic structure may cause effective information to be drowned out by noise, hinder the dissemination of effective information, and thereby increase the difficulty of collaborative governance. In contrast, except for Scenario 3, the other governance strategies delay the peak of structural disorder and significantly reduce the entropy peak, implying a higher degree of system order. This mitigation of structural instability provides a more favorable temporal and environmental window for the release and clarification of official information. Specifically, Scenarios 1 and 4 maintain relatively lower entropy levels, indicating that reducing the contact rate and lowering individual psychological involvement can enhance structural order in the system. For Scenario 2, previous analysis demonstrated that weakening herd behavior effectively suppresses the spread of public opinion. However, its entropy peak remains relatively high. This phenomenon can be explained by the fact that while weakening herd behavior restricts the occurrence of large-scale cascade effects in public opinion dissemination, it also stimulates more independent and diversified individual decision-making. Such changes further lead to a more dispersed distribution of public attitudes and behaviors, thereby increasing the structural uncertainty of the system. These indicate that the relationship between entropy and governance effectiveness is complex, and maintaining order in the system is not necessarily the governance goal of public opinion. Therefore, policymakers need to balance two objectives: limiting propagation intensityand managing structural uncertainty, recognizing that lower entropy does not automatically imply optimal governance outcomes. Nevertheless, the structural disorder of the public opinion system reflected by the differences in entropy levels and peak values under different scenarios can specifically guide policymakers in formulating and adjusting intervention strategies. Finally, it should be noted that the model assumes a continuous inflow of new individuals into the system, which may cause the aggregate population proportions to exceed 1. Therefore, we focus on the time interval [0,30] for analysis to illustrate the relationship between system entropy and public opinion governance.

To clarify the practical relevance of the sensitivity and entropy analyses for policymakers, Table 6 summarizes the changes in spreader peaks and entropy peaks relative to the baseline scenario, together with the associated governance strategies. Scenario 3 isn’t discussed in Table 6, because its governance effect is unsatisfactory. This also verifies the previous theoretical analysis, that is, the parameter corresponding to Scenario 3 is not involved in the expression of the propagation threshold, and is not a key parameter.

The primary objective of public opinion governance is to reduce the number ofspreaders, facilitate the effective dissemination of authoritative information, and guide the system toward a stable state. Accordingly, effective interventions should be capable of altering the structural proportions of the S, I, and R, thereby decreasing the number of spreaders and promoting convergence of the system to stability. From Table 6, the following conclusions can be drawn. First, controlling the transmission coefficient can significantly reduce both the peak of spreaders and the peak of system entropy, while also noticeably delaying their occurrence. This indicates that reducing contact rates, mitigating the perceived severity of the disaster, and regulating psychological involvement are structurally effective governance strategies. Such measures weaken the fundamental drivers of information propagation, enabling the system to converge more rapidly toward a stable state. Second, suppressing herd behavior can effectively lower the peak of spreaders, yet in some scenarios its impact on entropy is limited. Interventions targeting herd behavior tend to increase diversity of individual opinions, which may challenge system stability. Therefore, herd behavior mitigation is better suited as a supplementary strategy rather than a core governance approach. Third, reducing public psychological involvement exhibits strong entropy-controlling capability. Involvement not only affects whether individuals participate in information propagation but also influences the likelihood of spreaders converting to stiflers. Measures such as emotional cooling, timely dissemination of authoritative information, and risk perception guidance can decrease psychological involvement, simultaneously weakening propagation intensity and reducing system uncertainty.

## 6. Managerial Implications

Based on theoretical and simulation analyses, as well as case studies, the strategies and recommendations for controlling public opinion dissemination in human-made disasters are summarized as follows:

(1) Reduce the basic intensity of propagation from the source through timely responses.

The analyses indicate that the transmission coefficient is a key parameter of the propagation threshold, and reducing the transmission coefficient can simultaneously lower both the peak of spreaders and the peak of system entropy, while noticeably delaying their occurrence. This suggests that weakening the propagation force from the source is the most structurally effective governance strategy. In human-made disasters, the public exhibits a high demand for accurate information. Delayed official responses create an information vacuum, which can rapidly amplify propagation intensity and increase system uncertainty. Therefore, relevant authorities should promptly initiate investigations, release timely updates on event progress, and actively address public concerns. This approach meets the public’s demand for information while effectively limiting the space for rumor propagation. By shortening the information gap, contact rates and emotional amplification effects are reduced, weakening the conversion of ignorants into spreaders and allowing the system to converge to stability more quickly.

(2) Mitigate psychological involvement through the media.

Psychological involvement not only determines whether individuals participate in propagation but also affects the probability of spreaders converting into stiflers. Reducing psychological involvement can significantly decrease the number of spreaders and exhibits strong control over system entropy. In human-made disaster events, emotions such as anger, panic, and moral condemnation often accumulate rapidly, pushing the system into a high-uncertainty state. Therefore, mainstream media and authoritative platforms should not only follow up on factual developments but also assume responsibilities for emotional regulation and agenda guidance. By providing rational analyses, and professional interpretations, public emotions can be redirected toward reasoned discussion, thereby lowering psychological involvement. As involvement decreases, individuals’ sustained attention to information diminishes, propagation motivation declines, and spreaders more readily convert into stiflers, resulting in a reduction in system entropy. In this sense, mitigating psychological involvement serves as a critical bridge linking individual-level psychological regulation with group-level structural optimization.

(3) Moderate intervention on herd behavior to prevent persistent structural disorder.

Results also show that suppressing herd behavior can effectively reduce the peak of spreaders; however, in some scenarios, its impact on system entropy is limited. This indicates that merely weakening group-following behavior does not fundamentally alter the structural state of the public opinion system. In reality, especially within human-made disasters, public discourse is prone to losing focus and being over-interpreted, for example, speculations on motives, extended discussions that deviate from the core issue. Excessive suppression of herd behavior may increase information diversity, and further disperse the state of the public opinion system. Therefore, governance of herd behavior and off-topic discourse should prioritize clarifying agenda boundaries and regulating information sources rather than simply restricting expression.

(4) Collaborative governance of disaster management and public opinion guidance.

In complex network environments, public opinion involves diverse actors and multi-polar dissemination pathways, making it difficult for a single entity to achieve effective control. Only through coordinated mechanisms among the government, mainstream media, and the public can the system structure be effectively optimized. In human disasters, the government should strengthen authoritative information release, disaster management and online governance capacities; media should enhance agenda-setting and guiding capabilities, and guide the public’s emotions and focus of attention; and the public should improve media literacy and critical skills. By integrating mainstream media and self-media resources and implementing strategies that modify propagation dynamics and psychological drivers, authorities can achieve orderly guidance within an open public discourse environment.

## 7. Discussion

This study develops a public opinion propagation model for human-made disasters based on the classical SIR epidemic model, considering psychological involvement and conformity behavior. The model is empirically validated using the case of the ‘the Dalian intentional vehicle-ramming incident motivated by retaliation against society’. The results indicate that the transmission rate, suppression rate, and herd behavior coefficient are critical parameters influencing the propagation threshold of public opinion. These factors play decisive roles in determining the diffusion scale, peak value, and the evolutionary trajectory of system entropy. Simulation findings further demonstrate that psychological involvement and herd behavior exert substantial influence during the propagation process. Their impact on the proportion of spreaders exceeds that of the contact rate. However, after controlling for herd behavior, the variation in system entropy becomes relatively limited. These findings suggest that public opinion systems are not governed solely by the intensity of information exposure; rather, they are more fundamentally shaped by individual psychological involvement and collective interaction mechanisms. Unlike previous studies that primarily focused on the disaster event itself, this research highlights the distinctive characteristics of public opinion dynamics in human-made disasters by explicitly incorporating psychological involvement and herd behavior. The proposed framework extends the explanatory boundary of the SIR model in social information systems, advancing its applicability toward psychologically driven social diffusion processes.

This study finds that herd behavior can expand the scale of public opinion diffusion, which is consistent with the conclusions of existing relevant studies [69]; in addition, the herd effect can significantly accelerate information dissemination [62]. However, in contrast to the model proposed by Yan et al. [63], our results suggest that even in the presence of herd behavior, the system can converge to a stable equilibrium under certain conditions, rather than exhibiting persistent oscillations or uncontrolled diffusion. This point has been demonstrated theoretically and through simulations in this study. Furthermore, the findings indicate that weakening herd behavior, although effective in reducing the peak proportion of spreaders, may sustain a relatively high level of system entropy. This phenomenon implies that excessive emphasis on individual independence may lead to a more fragmented information structure, thereby increasing structural complexity and the risk of systemic disorder. Therefore, public opinion governance in practice should strike a balance between suppressing herd behavior and maintaining structural order. Consistent with previous studies [70,71], the results confirm that the transmission rate remains one of the primary determinants of propagation dynamics, and reducing contact between spreaders and ignorants continues to be an effective strategy for controlling public opinion diffusion. Unlike many existing disaster-related public opinion models that pay limited attention to individual psychological involvement, this study highlights the critical role of this psychological variable in shaping public opinion evolution during disasters. Both model analysis and case validation demonstrate that psychological involvement not only significantly affects the number of spreaders but also substantially influences system entropy, underscoring the importance of individual psychological factors in the evolution of public opinion. Empirical evidence from prior studies likewise confirms the significant effect of involvement on users’ reposting behavior [72].

Public opinion diffusion inherently occurs in complex network, so the findings of this study can also be interpreted from the perspective of network-based intervention theory. Network-based intervention research emphasizes optimizing system performance by adjusting node connections or influencing the behavior of key nodes. In the proposed model, the regulation of transmission rate, psychological involvement, and herd behavior corresponds to interventions on edge weights, node state transition probabilities, and collective imitation mechanisms in the network. From the perspective of community network intervention, opinion leaders or highly connected nodes exert amplification effects in the process of public opinion dissemination. Reducing psychological involvement and moderating herd behavior effectively weakens the reinforcement mechanisms and collective emotions, thereby mitigating the clustering of opinions and emotions in subnetworks. Accordingly, the proposed model provides parameter guidance for precision intervention strategies grounded in network structure. This analysis suggests that public opinion governance is not merely an issue of information release, but rather a matter of coordinated regulation between network structure and psychological mechanisms. By integrating structural interventions with psychological regulation strategies, more robust and stable system control can be achieved.

This study provides a quantitative foundation for governments and social media platforms in formulating public opinion guidance strategies. The results indicate that, compared with merely reducing the contact rate, interventions targeting psychological involvement and herd behavior exert a deeper and more fundamental influence on the diffusion process, having a substantially greater impact on the size of spreaders and system stability. This suggests that in a highly developed social media environment, emotional regulation, agenda guidance, and the establishment of rational deliberation mechanisms are more critical than simple information control. However, weakening herd behavior enhances individual independence and behavioral heterogeneity, potentially resulting in a higher-entropy and more disordered system. Therefore, conformity behavior should be guided appropriately rather than suppressed indiscriminately. At the practical level, these findings imply that when responding to public opinion crises triggered by human-made disasters, relevant authorities should adopt a comprehensive governance approach that integrates internal psychological guidance with external structural interventions, rather than relying solely on information blocking or suppression strategies.

This study conducts an empirical analysis based on Weibo data and validates the findings through a combination of theoretical modeling and simulation results. Nevertheless, several limitations should be acknowledged. First, although the sample ensured the user’s account activity, potential biases may exist in terms of age structure and regional distribution. In addition, it is difficult to directly identify from textual data whether individuals’ posting behaviors are driven primarily by rational judgment or by herd behavior. This unobservability may affect the estimation of certain model parameters. Second, the analysis is based on a single human-made disaster case. The selected case is representative and exhibits typical characteristics of public opinion dynamics in such events, thereby facilitating an in-depth exploration of the propagation mechanism, but different disasters may vary in evolution process. Therefore, future research could incorporate experimental or survey data to further examine the micro-level mechanisms of psychological involvement and herd behavior. Moreover, comparative analyses across multiple cases and platforms would help assess the robustness and generalizability of the proposed model across different disasters.

## 8. Conclusions

This study addresses the propagation of online public opinion in human-made disasters. Considering the distinctive characteristics of such disasters, it integrates both internal and external factors influencing individuals’information-sharing behavior, and two key factors, psychological involvement and herd behaviorinto a variants of SIRpropagation model. Through theoretical analysis, the public opinion propagation threshold was derived, revealing that transmission rate, stifling rate, and herd behavior coefficient are the critical parameters affecting this threshold. The simulation results validate the theoretical findings and provide a visualization of the sensitivity of the key parameters. Empirical analysis based on the Dalian intentional vehicle-ramming incident, motivated by retaliation against society, validated the effectiveness of the model. The results indicate that controlling contact rates, reducing herd behavior, and lowering individual psychological involvement level can effectively regulate public opinion, with the latter two factors proving more effective than controlling contact rates alone. Furthermore, this study analyzes the information entropy of group states and explores the relationship among the peak number of spreaders, governance effects, and information entropy. Accordingly, measures taken by the government and the media to alleviate public psychological involvement and herd behavior constitute an effective strategy to curb the spread of public opinion, but reducing herd behavior makes the system relatively more uncertain compared with other scenarios. Based on theoretical analysis, simulation analysis, and case analysis, managerial implications for public opinion governance of man-made disasters are proposed. This study not only provides theoretical support for understanding the evolution mechanisms of public opinion in human-made disasters but also offers empirical evidence for governments and social media platforms to develop scientific and rational public opinion management. Future research could consider individual heterogeneity to model, thereby enhancing its predictive capacity and applicability to complex public opinion events.

## Figures and Tables

**Table 1:** Description of parameters.

Parameters Term	Description
a Inflow rate	Proportion of individuals entering the virtual population per unit time
λ Contact rate	Probability per unit time that an ignorant individual contacts a spreader and becomes spreaders
D Disaster severity	Quantitative measure of the severity of the human-made disaster
P Involvement level	Quantitative measure of public involvement or Psychological involvementafter the disaster
b Transmission rate	Probability per unit time that an ignorant individual acquires public opinion information after contact with a spreader
c Outflow rate	Proportion of individuals leaving the virtual population per unit time
h Immunity rate	Probability that spreaders do not transmit information and become stiflers
S Ignorants	Proportion of netizens in the population who have never received any public opinion information. Ignorants have no ability to inhibit the spread of online public opinion
I Spreaders	Proportion of netizens in the population who are aware of and actively disseminate public opinion information
R Stiflers	Proportion of netizens in the population who are aware of the information but are not interested in further spreading it
$b_{0}$ Herd behavior coefficient of spreaders	The coefficient of the first term in the Taylor expansion of the spreaders’ herd behavior function.
$b_{1}$ Herd behavior coefficient of spreaders	The coefficient of the second term in the Taylor expansion of the spreaders’ herd behavior function.
$c_{0}$ Herd behavior coefficient of stiflers	The coefficient of the first term in the Taylor expansion of the stiflers’ herd behavior function.
$c_{1}$ Herd behavior coefficient of stiflers	The coefficient of the second term in the Taylor expansion of the stiflers’ herd behavior function.

**Table 2:** Number of Weibo Posts per day.

Day	21 May	22 May	23 May	24 May	25 May	26 May	27 May
Post Number	0	8112	10,494	2007	529	358	1915
Day	28 May	29 May	30 May	31 May	1 June	2 June	3 June
Post Number	187	814	1200	173	59	36	29
Day	4 June	5 June	6 June	7 June	8 June	9 June	10 June
Post Number	35	86	75	89	60	63	36

**Table 3:** Summary of Sampled Users and Their Weibo Posts.

Day	22 May	23 May	24 May	25 May	26 May	27 May
User Number	149	277	115	41	38	172
Post Number	212	567	204	60	43	244
Day	28 May	29 May	30 May	31 May	1 June	
User Number	26	34	24	12	3	
Post Number	37	47	36	13	4	

**Table 4:** Model parameters for the case study.

State	Initial Population	Parameter Values	Number of State Transitions	Parameter Values
Ignorant	376	$S_{0}$ = 0.987	277	b = 0.5
Spreader	4	$I_{0}$ = 0.013	201	h = 0.53
stifler	0	$R_{0}$ = 0		

**Table 5:** Parameter settings for each simulation scenario.

Scenarios	$\bar{b}$	b	$\bar{b_{0}}$	$b_{0}$	$\bar{b_{1}}$	$b_{1}$	$\bar{h}$	h	Remarks
Basic Scenario	0	0.5	0	0.34	0	0.1	0	0.53	Basic
Scenario1	−0.2	0.3	0	0.34	0	0.1	0	0.53	Examine *b*
Scenario2	0	0.5	−0.2	0.14	0	0.1	0	0.53	$\mathrm{Examine}\;b_{0}$
Scenario3	0	0.5	0	0.34	−0.05	0.05	0	0.53	$\mathrm{Examine}\;b_{1}$
Scenario4	0	0.5	0	0.34	0	0.1	0.2	0.73	Examine h

**Table 6:** Relative changes in spreader peak and entropy compared to the basic scenario.

Scenario	Peak of Spreaders	Peak of Entropy	Governance Strategy
Scenario 1	Decreased and significantly delayed	Significantly decreased and significantly delayed	Reducing contact rate, lowering psychological involvement, and mitigating perceived disaster severity
Scenario 2	Significantly decreased and slightly delayed	Decreased and significantly delayed	Intervention at the group level to suppress herd amplification effects
Scenario 4	Significantly decreased and slightly delayed	Significantly decreased and significantly delayed	Reduce the public’s emotional intensity, attention, and psychological involvement

## Data Availability

All data sets used in this research can be found on https://pan.cdut.edu.cn/link/AA498BCE0E67384989BACA6CFD3DAB21D8 (accessed on 5 March 2026).
